# Different feedback modes of brain–computer interface training for upper limb motor function after stroke: a protocol for a systematic review and network meta-analysis

**DOI:** 10.3389/fneur.2026.1865968

**Published:** 2026-07-29

**Authors:** Yaojiang Li, Yunhong Deng, Lixia Deng, Xiao Lu, Congping Huang, Xing Chen, Yunyi Liu, Peiling Chen, Mingjun Tang, Fuqiang Wang

**Affiliations:** 1 Department of Rehabilitation Medicine, Guangdong Sanjiu Brain Hospital, Guangzhou, Guangdong, China; 2 Department of Rehabilitation Therapy, Guangdong Sanjiu Brain Hospital, Guangzhou, Guangdong, China; 3 College of Acupuncture and Rehabilitation, Guangzhou University of Chinese Medicine, Guangzhou, Guangdong, China

**Keywords:** brain–computer interface, feedback mode, network meta-analysis, stroke, study protocol, systematic review, upper limb function

## Abstract

**Background:**

Upper limb motor impairment after stroke is a major contributor to long-term disability and can have lasting effects on patients’ activities of daily living, social participation, and quality of life. Brain-computer interface (BCI) training establishes a closed-loop training process that links central nervous system activity, external feedback, and sensory reafferent input, and is considered a promising approach to facilitating neuroplastic reorganization and motor recovery. Existing studies on upper-limb rehabilitation after stroke have investigated several BCI feedback modes. However, the relative efficacy of these feedback modes has not been systematically compared across available randomized controlled trials.

**Methods:**

This study protocol has been registered in PROSPERO with registration number CRD420261370474 and will be conducted in accordance with the PRISMA-P statement and the PRISMA-NMA extension. PubMed, Embase, Web of Science, the Cochrane Library, and Scopus will be systematically searched from inception to March 2026. Only parallel-group randomized controlled trials will be eligible for inclusion. Participants will be patients with post-stroke upper-limb motor impairment. The experimental interventions will consist of BCI training with different feedback modes, and intervention nodes will be defined according to specific feedback categories, including functional electrical stimulation, robot- or exoskeleton-assisted feedback, visual feedback, virtual reality feedback, and combined multimodal feedback. Control groups will include conventional rehabilitation, sham BCI, no additional intervention, and other active interventions. The primary outcome will be the Fugl-Meyer Assessment for Upper Extremity (FMA-UE) score. The secondary outcomes will be the Action Research Arm Test (ARAT) score and the Wolf Motor Function Test (WMFT) score. Two reviewers will independently perform study selection, data extraction, and risk-of-bias assessment, and any disagreements will be resolved through discussion or adjudication by a third reviewer. If the evidence network is sufficiently connected, a network meta-analysis will be performed to compare the relative efficacy across different intervention nodes. Local inconsistency will be assessed using the node-splitting method, and the surface under the cumulative ranking curve (SUCRA) will be used to rank different feedback modes according to efficacy.

**Conclusion:**

This protocol specifies the methods for comparing different BCI feedback modes in post-stroke upper-limb rehabilitation and provides a transparent framework for the planned systematic review and network meta-analysis.

**Systematic review registration:**

https://www.crd.york.ac.uk/prospero/, identifier CRD420261370474.

## 1 Introduction

Stroke remains one of the leading causes of death and disability worldwide. With the increasing number of people living with stroke, long-term functional impairment and rehabilitation needs pose an increasingly important clinical and public health burden (1). Motor impairment is a major contributor to activity limitations and reduced quality of life after stroke. Upper limb dysfunction commonly involves impaired control of the shoulder, elbow, wrist, and hand, leading to reduced ability in grasping, object manipulation, self-care, and social participation. Previous systematic reviews have shown substantial interindividual variability in motor recovery after stroke, and the severity of initial motor deficits is an important factor associated with recovery outcomes; complete motor recovery is uncommon among patients with marked paresis (2). In addition, stroke recovery follows a distinct temporal course and involves different stages. Neuroplastic potential and responsiveness to intensive training may differ across these stages. Therefore, more effective and phase-specific rehabilitation strategies for upper limb dysfunction after stroke warrant further development and evaluation (3, 4).

Brain–computer interface (BCI) technology is rapidly evolving in neurorehabilitation. The basic principle is to acquire and decode brain activity signals associated with motor intention and translate them into control commands for external devices or sensory feedback, thereby establishing a closed-loop training process that links motor intention, external feedback, and sensory reafferent input (5–7). Compared with conventional rehabilitation approaches that mainly rely on repetitive peripheral practice, BCI-based rehabilitation training places greater emphasis on detecting patients’ active motor intention and on the temporal coupling of task-related brain activity with external feedback. Therefore, BCI-based rehabilitation is considered a promising approach for promoting motor learning and neuroplastic reorganization through closed-loop feedback mechanisms (5–7). In patients with limited residual motor ability or even no overt voluntary movement, BCI can still detect brain activity associated with motor imagery or attempted movement to guide external feedback or assisted movement, suggesting its considerable potential for upper limb rehabilitation after stroke (6, 7).

BCI training should not be regarded as a homogeneous intervention. Existing studies have commonly combined BCI with various external feedback modalities or effector devices, including functional electrical stimulation (FES), robotic or exoskeleton-assisted systems, visual/virtual reality feedback, and combined multimodal feedback (7–10). Because these feedback modalities and effector devices differ in terms of sensory afferent input, motor assistance, task immersion, and closed-loop feedback pathways, their underlying mechanisms of action and rehabilitation effects may also vary (7–10). For example, BCI-FES may couple movement intention-related brain activity with electrical stimulation of the target muscles and the resulting limb movement, thereby generating peripheral feedback that is matched to attempted movement and potentially promoting reorganization of motor-related neural networks (7, 11). Robot- or exoskeleton-assisted approaches may use decoded brain signals to deliver repetitive, goal-directed motor assistance and movement-related feedback to patients (8). Visual or virtual reality feedback may provide action observation, real-time feedback, and immersive task contexts during BCI-based motor imagery training, which may help enhance training engagement and the quality of motor representation (9). Combined multimodal feedback may further reinforce the sensorimotor closed loop during motor imagery training by integrating multisensory information, including visual, tactile, and proprioceptive inputs (10). However, existing evidence has largely focused on specific BCI feedback forms or individual technical combinations, and the comparative clinical advantages of different feedback modes remain insufficiently investigated.

Randomized controlled trials (RCTs) and conventional meta-analyses have suggested that BCI training may improve upper limb motor function after stroke (12–14). However, previous evidence has largely been based on pairwise comparisons of BCI training with conventional rehabilitation or other control interventions. Although some studies have examined whether BCI design characteristics influence treatment effects, the relative efficacy and ranking of BCI training with different feedback modes have not been systematically assessed within a unified statistical framework. Moreover, variations in BCI design characteristics, feedback approaches, control conditions, disease stage, and training dose across previous studies limit the ability of traditional pairwise comparisons alone to provide sufficient evidence for selecting BCI feedback modes in clinical practice (12, 13).

Network meta-analysis (NMA) can integrate direct and indirect evidence within a unified statistical framework, evaluate the relative efficacy of multiple interventions, and provide rankings of treatment effects. Therefore, NMA is particularly appropriate for the current evidence landscape in brain-computer interface (BCI)-based rehabilitation, which is characterized by multiple intervention types and limited direct head-to-head evidence (15). Accordingly, this study will conduct a systematic review and NMA to compare the relative efficacy of BCI training with different feedback modes for upper limb motor function after stroke, with prospective registration and standardized methods used to enhance the transparency and credibility of the review.

## 2 Methods and analysis

### 2.1 Study registration and reporting guidelines

This protocol describes a systematic review and NMA and has been registered with the International Prospective Register of Systematic Reviews (PROSPERO), under registration number CRD420261370474. This protocol was prepared and is reported in accordance with the relevant requirements of the Preferred Reporting Items for Systematic Review and Meta-Analysis Protocols (PRISMA-P). The subsequent systematic review and NMA will be reported in accordance with the relevant principles of the PRISMA extension statement for network meta-analyses (PRISMA-NMA) (16, 17). During the conduct of the review, any major amendments to key elements of the registered protocol, including eligibility criteria, intervention-node classification, outcome definitions, or statistical analysis methods, will be described in the final review report to enhance the transparency and traceability of the research process.

### 2.2 Inclusion and exclusion criteria

This study will include only parallel-group RCTs. Only peer-reviewed, full-text journal articles published in English will be eligible. Conference abstracts, dissertations, theses, and other grey literature will be excluded. Eligible participants will be patients with clinically confirmed or neuroimaging-confirmed stroke and varying degrees of upper limb motor impairment. No restrictions will be imposed on age, sex, stroke type, lesion location, or post-stroke stage. The experimental intervention must be BCI-based upper limb rehabilitation training, defined as a closed-loop training process that uses electroencephalographic signals or other brain activity signals as input and provides feedback through an external feedback device or system. Control interventions may include conventional rehabilitation, usual care, sham BCI, no additional intervention, or other active control interventions. Studies comparing two or more BCI interventions with different feedback modes will also be included to increase direct comparative evidence within the network.

During full-text assessment, studies will be excluded if they meet any of the following criteria: (1) the study involves a stroke rehabilitation intervention but is not a parallel-group RCT, including crossover studies, non-randomized controlled studies, and observational studies; (2) the participants are not patients with stroke, or the study includes both patients with stroke and patients with other neurological disorders, but data for patients with stroke cannot be extracted separately; (3) the intervention is not BCI-based upper limb rehabilitation training, or the BCI feedback mode cannot be clearly determined from the original article; (4) BCI training is combined with additional neuromodulation or other intensified co-interventions, and the independent effect of BCI feedback training cannot be isolated; (5) the study does not report the Fugl-Meyer Assessment for Upper Extremity (FMA-UE) score, or outcome data suitable for quantitative analysis cannot be obtained; and (6) in cases of duplicate publication or overlapping sample data, the version with the most complete information, the clearest outcome reporting, or the largest amount of usable sample data will be retained.

Crossover RCTs will be excluded because BCI-based rehabilitation training may induce motor learning and neuroplastic changes that persist beyond the intervention period, making carryover effects difficult to rule out. In addition, post-stroke motor recovery changes over time, which may introduce period effects and confounding by spontaneous recovery.

Studies combining BCI training with additional neuromodulation or other intensified co-interventions will be excluded if the independent effect of BCI feedback training cannot be isolated. This criterion is intended to avoid confounding between the effect of the BCI feedback mode and that of additional co-interventions. If an intervention uses a BCI-contingent output that falls within the predefined feedback-mode classification and does not introduce an independent add-on treatment effect, it will be assessed according to the prespecified node classification rules.

The primary outcome will be the FMA-UE score. The secondary outcomes will be the Action Research Arm Test (ARAT) score and the Wolf Motor Function Test (WMFT) score. If a study reports multiple assessment time points, the primary analysis will preferentially use the change from baseline at the end of the intervention. Information from other time points may be recorded but will not be included in the primary quantitative synthesis. Studies will not be excluded solely because they do not report the secondary outcomes. The PICOS framework is presented in Table 1.

**Table 1:** PICOS framework of the study.

Item	Description
Population	Patients with clinically diagnosed or imaging-confirmed stroke and upper limb motor dysfunction
Intervention	BCI-based upper limb rehabilitation training will be categorized according to feedback mode as BCI-FES, BCI-robot, BCI-visual, BCI-VR, BCI-multimodal, or other BCI-based feedback modes.
Comparator	Conventional rehabilitation, usual care, sham BCI, no additional intervention, or other active control interventions
Outcome	Primary outcome: FMA-UE score. Secondary outcomes: ARAT score and WMFT score.
Study design	Parallel-design RCTs

### 2.3 Principles for classifying intervention nodes

As this study aims to compare the relative efficacy of BCI interventions with different feedback modes, interventions will be classified into network nodes according to prespecified rules before statistical modeling. Studies in which brain signals trigger or modulate FES as the primary feedback modality will be assigned to the BCI-FES node. Studies in which robots, robotic arms, hand exoskeletons, soft robotic gloves, or other mechanically assisted devices serve as the primary feedback modality will be assigned to the BCI-robot node. Studies in which non-immersive visual displays, such as screen cursors, virtual hands, movement animations, or other monitor-based visual feedback, are used as the primary feedback modality will be assigned to the BCI-visual node. Studies in which virtual reality interfaces, VR scenes, or virtual task environments are used as the primary feedback modality will be assigned to the BCI-VR node. If an intervention combines two or more feedback modalities and no single feedback mode can be clearly identified as dominant, it will be assigned to the BCI-multimodal node.

In addition to the experimental intervention nodes described above, control conditions such as motor imagery training alone, FES alone, robot-assisted training alone, conventional rehabilitation, or sham BCI will be treated as separate nodes according to their clinical relevance and network-structure requirements. Node classification will be independently determined by two reviewers on the basis of the intervention descriptions in the original studies. Any disagreements will be resolved through discussion, with a third reviewer consulted when necessary. This node classification strategy is intended to balance clinical interpretability with network connectivity, thereby supporting the plausibility of indirect comparisons to the extent possible. The definitions and classification criteria for each intervention node are presented in Table 2.

**Table 2:** Principles for defining and classifying intervention nodes.

Node name	Definition	Typical form
BCI-FES	BCI interventions in which brain signals trigger or modulate FES as the primary feedback modality	EEG-BCI–triggered FES targeting the wrist or finger extensors
BCI-robot	BCI interventions that primarily use mechanical assistive devices, such as robotic arms, exoskeletons, or soft gloves, as feedback mechanisms	Training with BCI-controlled exoskeletons or robotic arms
BCI-visual	BCI interventions that use non-immersive, monitor-based visual displays as the primary feedback modality	Screen cursors, two-dimensional movement animations, screen-based virtual hand animations, or other non-immersive visual feedback
BCI-VR	BCI interventions that use virtual reality interfaces or virtual task environments as the primary feedback modality	VR scenes, immersive or semi-immersive virtual environments, or VR-based task environments
BCI-multimodal	BCI interventions that integrate two or more feedback modalities without a single dominant modality	BCI plus FES and visual feedback, or BCI plus robotics and visual feedback
Usual rehabilitation	Conventional rehabilitation or usual care without BCI-based feedback	Physical therapy, occupational therapy, conventional upper-limb training, or routine rehabilitation care
sham BCI	Fake BCI or non-contingent BCI feedback	Fake feedback or pseudo-random feedback
FES	Standalone FES training without BCI	Conventional FES
Robot	Training using robots or exoskeletons alone without BCI	Robot-assisted therapy
MI	Motor imagery training without BCI devices	Motor imagery training

### 2.4 Information sources and search strategy

This study will systematically search PubMed, Embase, Web of Science, the Cochrane Library, and Scopus from their inception to March 2026. The search strategy will be developed using a combination of controlled vocabulary and free-text terms related to stroke, brain–computer interface, upper limb function, and randomized controlled trials. Key search terms will include, but will not be limited to, “stroke,” “brain–computer interface,” “BCI,” “upper extremity,” and “randomized controlled trial.” The search strings will be adapted to the indexing system and syntax of each database. The detailed search strategy will be provided in Supplementary File S1.

### 2.5 Study selection process

**Figure 1:**
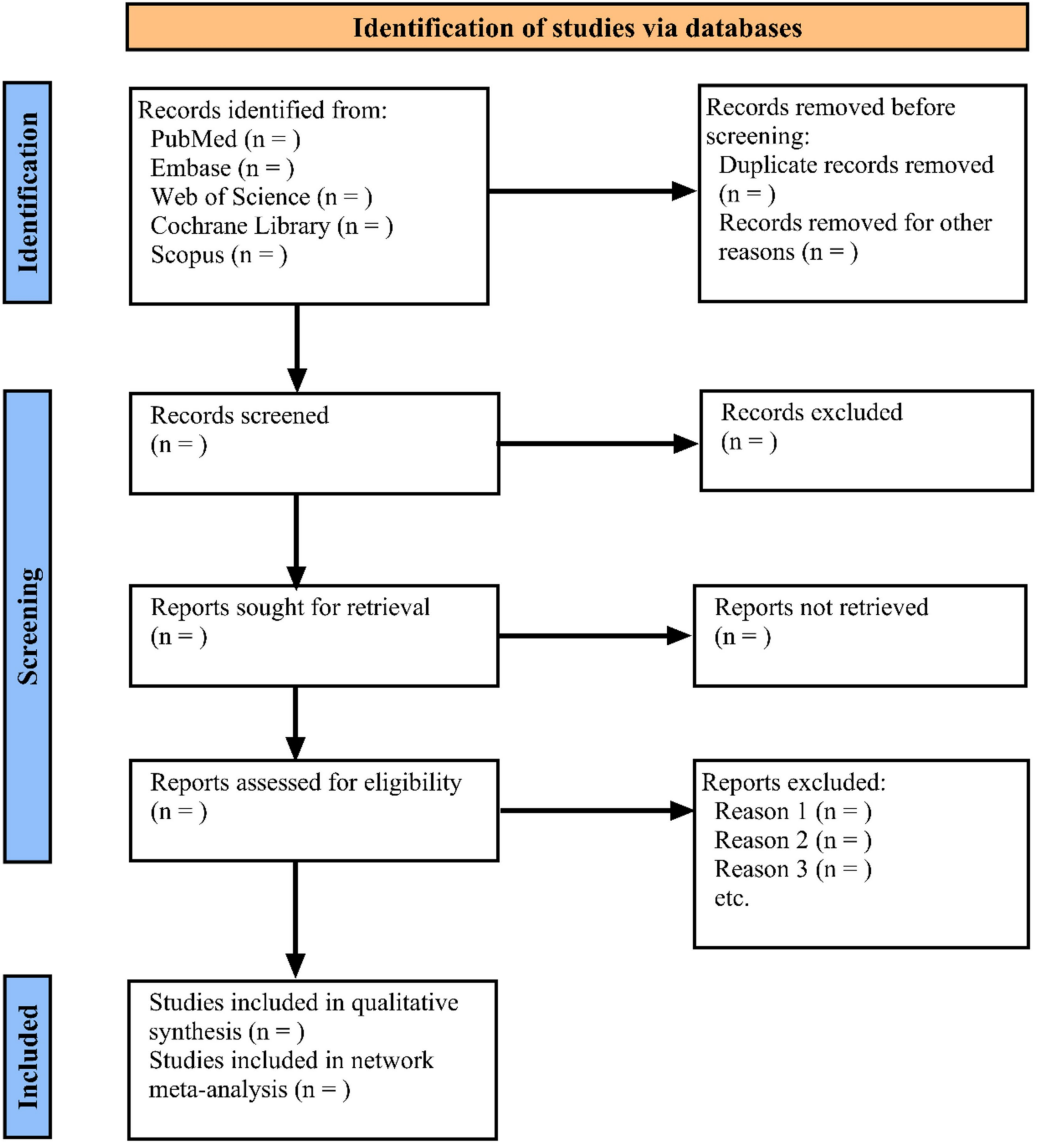
Planned PRISMA 2020 flow diagram for study selection. The final numbers of records and reports, reasons for exclusion, and studies included will be reported in the completed systematic review and NMA.

All records retrieved from the searches will be imported into reference management software, such as EndNote, NoteExpress, or equivalent software, for centralized management and duplicate removal. After deduplication, two researchers will independently screen the titles and abstracts of all records against the predefined inclusion and exclusion criteria. Records that cannot be clearly excluded on the basis of title and abstract screening will be retrieved in full text and assessed for eligibility. During full-text screening, two reviewers will independently assess each study against the eligibility criteria and record the specific reasons for excluding full-text articles. Any disagreements between the two reviewers during the screening process will first be resolved through discussion; unresolved disagreements will be adjudicated by a third reviewer. The study selection process will be presented using a PRISMA 2020 flow diagram, showing record identification, duplicate removal, screening, full-text assessment, exclusion, and final inclusion (Figure 1) (18).

### 2.6 Data extraction and data management

Two researchers (YJ and YH) will independently extract data using a predefined, pilot-tested data extraction form. The extracted information will include study characteristics, participant characteristics, intervention and comparator conditions, training parameters, primary outcome data, secondary outcome data, exploratory neurophysiological measures, and BCI performance metrics. Specifically, the following variables will be extracted: first author, publication year, country or region, sample size, patient age, post-stroke duration, detailed interventions in the experimental and control groups, BCI feedback mode, session duration, weekly training frequency, total treatment duration, and group-specific means, standard deviations, and sample sizes for the FMA-UE, ARAT, and WMFT, where available. Exploratory neurophysiological measures, including electroencephalography (EEG) changes, event-related desynchronization/synchronization (ERD/ERS) modulation, and related sensorimotor rhythm activity, will be extracted when reported in the included studies. BCI performance metrics, including classification accuracy, online control accuracy, task success rate, information transfer rate, the number or proportion of valid training trials or sessions, calibration performance, and classifier performance, will also be extracted when reported. These measures will be summarized descriptively because their definitions, acquisition methods, and reporting formats may differ across studies.

For studies with incomplete data or insufficiently reported statistics, the study authors will first be contacted to request missing information, where possible. If the standard deviation is not directly reported but the standard error, 95% confidence interval, or other statistics that can be converted are provided, standard conversion methods used in systematic reviews and meta-analyses will be applied. If continuous outcome data are reported as medians, ranges, or interquartile ranges, the mean and standard deviation will be estimated using established methods based on the sample size, median, range, and/or interquartile range (19). If outcome data are presented only in graphical form, appropriate digital extraction tools will be used to support data extraction when necessary. Any discrepancies between the two researchers during data extraction will first be resolved through discussion; if consensus cannot be reached, a third reviewer will adjudicate.

### 2.7 Risk-of-bias assessment

The risk of bias in the included RCTs will be assessed using the revised Cochrane risk-of-bias tool for randomized trials (RoB 2) (20). The RoB 2 tool evaluates outcome-specific risk of bias across five domains: the randomization process, deviations from intended interventions, missing outcome data, measurement of the outcome, and selection of the reported result. Based on judgments across these domains, an overall risk-of-bias judgment will be assigned and classified as “low risk,” “some concerns,” or “high risk” (20).

Two researchers (LX and XL) will independently assess the risk of bias and compare their judgments. Any disagreements will be resolved through discussion and, if necessary, through adjudication by a third reviewer. Given that complete blinding of participants and intervention providers is often difficult in rehabilitation studies, this review will apply the RoB 2 framework in relation to the specific design of each study. Particular attention will be paid to whether the randomization process is adequate, whether there is a risk of deviations from the intended interventions, whether outcome assessment is blinded, whether missing outcome data are appropriately handled, and whether prespecified outcomes are fully reported.

### 2.8 Statistical analysis

We will first conduct conventional pairwise meta-analyses for interventions with direct comparative evidence. If a connected evidence network can be established across intervention nodes, we will conduct an NMA to synthesize direct and indirect evidence within a unified statistical framework and compare the relative efficacy of BCI interventions with different feedback modes for post-stroke upper limb motor dysfunction (15, 21). If the evidence network is disconnected, NMA will not be performed; instead, pairwise meta-analyses or narrative synthesis will be undertaken according to the available evidence. All quantitative analyses will be performed using Stata and/or R.

The primary analysis will be based on a frequentist random-effects NMA model. Given the expected heterogeneity among included studies in stroke stage, baseline upper limb function, training dose, treatment duration, co-administered conventional rehabilitation, and control conditions, a random-effects model will be used to pool effect estimates. The transitivity assumption will be assessed descriptively by comparing the distributions of key potential effect modifiers across treatment comparisons. The following variables will be examined: stroke stage or post-stroke duration, baseline upper limb motor function, intervention dose, treatment duration, control condition type, and overall risk-of-bias judgment. Baseline upper limb motor function will be assessed primarily using baseline FMA-UE scores. Intervention dose will be assessed using the total number of training sessions, and treatment duration will be assessed in weeks, where available. Control condition type will be categorized as conventional rehabilitation or usual care, sham BCI, no additional intervention, or other active control interventions. Overall risk of bias will be classified as low risk, some concerns, or high risk according to the RoB 2 assessment. These characteristics will be presented in a structured summary table stratified by treatment comparison. Continuous variables will be presented as means and standard deviations or medians and interquartile ranges, as appropriate, and categorical variables will be presented as frequencies and proportions. Potentially important imbalance will be considered when continuous variables show clearly separated distributions across comparisons or marked differences in means or medians, or when the proportion of a categorical characteristic differs by more than 20 percentage points between comparisons. When such imbalance is identified, the potential impact on the validity of indirect comparisons will be discussed, and findings from indirect comparisons and treatment rankings will be interpreted with caution.

The primary outcome will be the FMA-UE score. The secondary outcomes will be the ARAT score and WMFT score. For continuous outcomes measured using the same scale, the effect size will be expressed as the mean difference (MD) with 95% confidence intervals (CIs). If different instruments are used to assess comparable upper limb functional outcomes, the standardized mean difference (SMD) with 95% CIs will be used for pooling. The ARAT and WMFT will be analyzed separately where appropriate. Secondary outcomes will be quantitatively synthesized when sufficient data and a connected evidence network are available; otherwise, pairwise meta-analysis or narrative synthesis will be performed according to the available evidence.

For multi-arm trials, all eligible intervention groups will be included in the NMA. For multiple effect estimates derived from the same study, the correlation among comparisons will be appropriately accounted for to avoid double-counting participants in shared control groups. In conventional pairwise meta-analyses, shared control groups will be split according to standard methods when necessary.

A network evidence plot will be used to display direct comparisons among intervention nodes. Node size will be proportional to the sample size of the corresponding intervention, and line thickness will be proportional to the number of studies directly comparing the interventions (21). The results of the NMA will be reported as relative effect estimates between interventions with 95% CIs.

Network consistency will be assessed using the node-splitting method to compare the agreement between direct and indirect evidence (22). If no substantial inconsistency is detected, the main results will be interpreted under the consistency model. If inconsistency is present, the results will be interpreted cautiously, with consideration of participant characteristics, intervention node classification, and the structure of the evidence network.

After the NMA is completed, the surface under the cumulative ranking curve (SUCRA) will be used to assess the relative ranking probabilities of different interventions (21, 23). A higher SUCRA value indicates that the intervention is more likely to rank among the best options. Ranking results will be interpreted alongside the magnitude of effect estimates, 95% CIs, precision of evidence, and network structure, rather than used alone to determine intervention superiority.

### 2.9 Assessment of heterogeneity

Between-study heterogeneity will be evaluated using Cochran’s Q test and the I^2^ statistic. The I^2^ statistic will be used to describe the proportion of total variation in effect estimates attributable to between-study heterogeneity rather than to sampling error (24). A higher I^2^ value indicates greater heterogeneity across studies; its interpretation will be based on the effect size, the direction of effects, the results of Cochran’s Q test, and the number of included studies. If sufficient data are available, subgroup analyses will be conducted to explore potential heterogeneity according to stroke stage or post-stroke duration, training dose, and control type. Stroke stage or post-stroke duration will be categorized according to the definitions reported in the original studies or, where applicable, as acute, subacute, or chronic stroke. Training dose will be assessed using the total number of training sessions and/or total treatment duration. Control type will be categorized as conventional rehabilitation or usual care, sham BCI, no additional intervention, or other active control interventions. Sensitivity analyses will be performed to examine the robustness of the findings by excluding studies at high risk of bias, studies with unclear or mixed stroke stage that cannot be classified, and studies with markedly different control conditions or training doses. These factors will be considered when interpreting heterogeneity and the robustness of the findings.

### 2.10 Assessment of small-study effects and potential publication bias

When a sufficient number of studies are available for the primary outcome, NMA–specific graphical methods, such as comparison-adjusted funnel plots, will be used as an exploratory assessment of small-study effects and potentially associated publication bias (23, 25). For direct comparisons with a sufficient number of included studies, conventional funnel plots will also be generated as a supplementary analysis to examine the distribution of effect sizes according to study precision. If clear asymmetry is observed in the plots, it will not be interpreted directly as evidence of publication bias. Instead, potential sources of asymmetry will be further explored by considering factors such as study size, publication status, study design, and methodological quality.

### 2.11 Assessment of the certainty of evidence

Based on the characteristics of the included studies, the structure of the evidence network, and the results of the primary outcome analyses, the certainty of evidence for the main comparisons will be assessed using the Grading of Recommendations Assessment, Development and Evaluation (GRADE) framework or the Confidence in Network Meta-Analysis (CINeMA) approach. The assessment will consider within-study bias, reporting bias, indirectness, imprecision, heterogeneity, and incoherence, and will integrate direct, indirect, and network estimates to inform the overall judgment. The certainty of evidence will be classified into four levels: high, moderate, low, and very low, reflecting the confidence in the evidence regarding the relative efficacy of different BCI feedback modes.

## 3 Discussion

In recent years, BCI technology has been increasingly applied in upper limb rehabilitation after stroke, and previous systematic reviews and meta-analyses have suggested its potential to improve upper limb motor function (12, 13). However, existing evidence has mainly evaluated whether BCI is superior to conventional therapy or whether specific BCI design characteristics modify treatment effects, whereas comparisons of the relative efficacy of different feedback modes remain limited (12, 13). From a clinical perspective, the key question is not only whether BCI is effective, but also which feedback modes are most likely to confer greater benefits for upper limb function when multiple feedback options are available.

A key feature of this protocol is that it considers “feedback mode” the core design factor and prespecifies intervention-node definitions at the outset of the study. By integrating different feedback modes, including BCI-FES, BCI-robot, BCI-visual, BCI-VR, and BCI-multimodal, within a common evidence network, this study aims to provide a clear methodological framework for comparing the relative efficacy of different BCI feedback modes. In addition, this study will include only parallel-group RCTs. Studies that combine BCI training with additional neuromodulation or other intensified co-interventions will be excluded if the independent effect of BCI feedback training cannot be isolated. This criterion is intended to reduce confounding arising from complex combined interventions when interpreting feedback-mode effects, thereby improving comparability across intervention nodes and enhancing the interpretability of the planned evidence synthesis.

This study has several potential limitations. First, included studies may differ in terms of disease duration, baseline function, training dose, concomitant conventional rehabilitation, and control conditions. These factors may influence intervention effects and contribute to clinical heterogeneity. Second, the number of studies evaluating some feedback modes may be limited, and certain comparisons may rely primarily on indirect evidence, which could affect the robustness of the conclusions. Third, to ensure clear node classification, this study will exclude studies involving complex combined interventions when the independent effect of BCI feedback training cannot be isolated. This approach may improve comparability across intervention nodes but may limit the generalizability of the planned review to settings involving complex combined interventions. Finally, the primary quantitative analysis will focus on changes in FMA-UE scores from baseline to the end of the intervention; therefore, assessment of long-term follow-up effects will be relatively limited.

Despite these limitations, this study protocol has methodological and clinical relevance. By prospectively specifying the search strategy, eligibility criteria, intervention-node classification, outcome definitions, data extraction rules, risk-of-bias assessment, and statistical analysis plan before completion of the review, this protocol provides a transparent methodological basis for the subsequent systematic review and NMA. This is particularly important for network meta-analysis, in which *post hoc* decisions regarding node classification, comparator handling, and analytical methods may influence the interpretation of comparative efficacy. In the future, as more RCTs directly compare different BCI feedback modes, the node classification and evidence synthesis framework established in this study may also inform subsequent updated reviews and clinical trial design.

## References

[1] GBD 2019 Stroke Collaborators. Global, regional, and national burden of stroke and its risk factors, 1990-2019: a systematic analysis for the global burden of disease study 2019. Lancet Neurol. (2021) 20:795–820. doi: 10.1016/s1474-4422(21)00252-0, 34487721 PMC8443449

[2] Hendricks HT van Limbeek J Geurts AC Zwarts MJ. Motor recovery after stroke: a systematic review of the literature. Arch Phys Med Rehabil. (2002) 83:1629–37. doi: 10.1053/apmr.2002.35473, 12422337

[3] Dromerick AW Geed S Barth J Brady K Giannetti ML Mitchell A . Critical period after stroke study (CPASS): a phase II clinical trial testing an optimal time for motor recovery after stroke in humans. Proc Natl Acad Sci USA. (2021) 118:e2026676118. doi: 10.1073/pnas.2026676118, 34544853 PMC8488696

[4] Ward NS. Restoring brain function after stroke - bridging the gap between animals and humans. Nat Rev Neurol. (2017) 13:244–55. doi: 10.1038/nrneurol.2017.34, 28303914

[5] Daly JJ Wolpaw JR. Brain-computer interfaces in neurological rehabilitation. Lancet Neurol. (2008) 7:1032–43. doi: 10.1016/s1474-4422(08)70223-0, 18835541

[6] Ushiba J Soekadar SR. Brain-machine interfaces for rehabilitation of poststroke hemiplegia. Prog Brain Res. (2016) 228:163–83. doi: 10.1016/bs.pbr.2016.04.020, 27590969

[7] López-Larraz E Sarasola-Sanz A Irastorza-Landa N Birbaumer N Ramos-Murguialday A. Brain-machine interfaces for rehabilitation in stroke: a review. NeuroRehabilitation. (2018) 43:77–97. doi: 10.3233/nre-172394, 30056435

[8] Baniqued PDE Stanyer EC Awais M Alazmani A Jackson AE Mon-Williams MA . Brain-computer interface robotics for hand rehabilitation after stroke: a systematic review. J Neuroeng Rehabil. (2021) 18:15. doi: 10.1186/s12984-021-00820-8, 33485365 PMC7825186

[9] Oliveira I Russo M Almeida AI Vourvopoulos A Mendes Pereira C. Recommendations for combining brain-computer interface, motor imagery, and virtual reality in upper limb stroke rehabilitation: qualitative participatory design study. JMIR Rehabil Assist Technol. (2025) 12:e71789. doi: 10.2196/71789, 41092418 PMC12527325

[10] Lu R Pang Z Gao T He Z Hu Y Zhuang J . Multisensory BCI promotes motor recovery via high-order network-mediated interhemispheric integration in chronic stroke. BMC Med. (2025) 23:380. doi: 10.1186/s12916-025-04214-8, 40598460 PMC12220625

[11] Biasiucci A Leeb R Iturrate I Perdikis S Al-Khodairy A Corbet T . Brain-actuated functional electrical stimulation elicits lasting arm motor recovery after stroke. Nat Commun. (2018) 9:2421. doi: 10.1038/s41467-018-04673-z, 29925890 PMC6010454

[12] Cervera MA Soekadar SR Ushiba J Millán JDR Liu M Birbaumer N . Brain-computer interfaces for post-stroke motor rehabilitation: a meta-analysis. Ann Clin Transl Neurol. (2018) 5:651–63. doi: 10.1002/acn3.544, 29761128 PMC5945970

[13] Mansour S Ang KK Nair KPS Phua KS Arvaneh M. Efficacy of brain-computer Interface and the impact of its design characteristics on poststroke upper-limb rehabilitation: a systematic review and Meta-analysis of randomized controlled trials. Clin EEG Neurosci. (2022) 53:79–90. doi: 10.1177/15500594211009065, 33913351 PMC8619716

[14] Pichiorri F Morone G Petti M Toppi J Pisotta I Molinari M . Brain-computer interface boosts motor imagery practice during stroke recovery. Ann Neurol. (2015) 77:851–65. doi: 10.1002/ana.24390, 25712802

[15] Rouse B Chaimani A Li T. Network meta-analysis: an introduction for clinicians. Intern Emerg Med. (2017) 12:103–11. doi: 10.1007/s11739-016-1583-7, 27913917 PMC5247317

[16] Shamseer L Moher D Clarke M Ghersi D Liberati A Petticrew M . Preferred reporting items for systematic review and meta-analysis protocols (PRISMA-P) 2015: elaboration and explanation. BMJ. (2015) 349:g7647. doi: 10.1136/bmj.g7647, 25555855

[17] Hutton B Salanti G Caldwell DM Chaimani A Schmid CH Cameron C . The PRISMA extension statement for reporting of systematic reviews incorporating network meta-analyses of health care interventions: checklist and explanations. Ann Intern Med. (2015) 162:777–84. doi: 10.7326/m14-2385, 26030634

[18] Page MJ McKenzie JE Bossuyt PM Boutron I Hoffmann TC Mulrow CD . The PRISMA 2020 statement: an updated guideline for reporting systematic reviews. BMJ. (2021) 372:n71. doi: 10.1136/bmj.n71, 33782057 PMC8005924

[19] Wan X Wang W Liu J Tong T. Estimating the sample mean and standard deviation from the sample size, median, range and/or interquartile range. BMC Med Res Methodol. (2014) 14:135. doi: 10.1186/1471-2288-14-135, 25524443 PMC4383202

[20] Sterne JAC Savović J Page MJ Elbers RG Blencowe NS Boutron I . RoB 2: a revised tool for assessing risk of bias in randomised trials. BMJ. (2019) 366:l4898. doi: 10.1136/bmj.l4898, 31462531

[21] Shim S Yoon BH Shin IS Bae JM. Network meta-analysis: application and practice using Stata. Epidemiol Health. (2017) 39:e2017047. doi: 10.4178/epih.e2017047, 29092392 PMC5733388

[22] Dias S Welton NJ Sutton AJ Caldwell DM Lu G Ades AE. Evidence synthesis for decision making 4: inconsistency in networks of evidence based on randomized controlled trials. Med Decis Mak. (2013) 33:641–56. doi: 10.1177/0272989x12455847, 23804508 PMC3704208

[23] Chaimani A Higgins JP Mavridis D Spyridonos P Salanti G. Graphical tools for network meta-analysis in STATA. PLoS One. (2013) 8:e76654. doi: 10.1371/journal.pone.0076654, 24098547 PMC3789683

[24] Higgins JP Thompson SG Deeks JJ Altman DG. Measuring inconsistency in meta-analyses. BMJ. (2003) 327:557–60. doi: 10.1136/bmj.327.7414.557, 12958120 PMC192859

[25] Chaimani A Salanti G. Using network meta-analysis to evaluate the existence of small-study effects in a network of interventions. Res Synth Methods. (2012) 3:161–76. doi: 10.1002/jrsm.57, 26062088

