# Supplementary material for: Different feedback modes of brain–computer interface training for upper limb motor function after stroke: a protocol for a systematic review and network meta-analysis
Source: Front Neurol. 2026 Jul 29;17:1865968. doi: 10.3389/fneur.2026.1865968 (PMC13461578; doi:10.3389/fneur.2026.1865968)
Supplement: Supplementary file 1 [file Data_Sheet_1.PDF]

Supplementary file 1: Search strategy

Electronic Databases: PubMed, Embase, Cochrane Library, Scopus and Web of Science.

1. PubMed Search Strategy:

#1 "Brain-Computer Interfaces"[Mesh]

#2 (((((((((((brain-computer interface[Title/Abstract]) OR (brain-computer interfaces[Title/Abstract])) OR (BCI[Title/Abstract])) OR (brain-machine interface[Title/Abstract])) OR (brain-machine interfaces[Title/Abstract])) OR (brain machine interface[Title/Abstract])) OR (brain machine interfaces[Title/Abstract])) OR (neural interface[Title/Abstract])) OR (neural interfaces[Title/Abstract])) OR (neural interface technology[Title/Abstract])) OR (neural interface technologies[Title/Abstract])) OR (brain signal interface[Title/Abstract])) OR (brain signal interfaces[Title/Abstract])

#3 #1 OR #2

#4 "Stroke"[Mesh]

#5 (((((((((((((((((((((((((((((strokes[Title/Abstract]) OR (acute strokes[Title/Abstract])) OR (acute stroke[Title/Abstract])) OR (cerebrovascular accident[Title/Abstract])) OR (cerebrovascular accidents[Title/Abstract])) OR (acute cerebrovascular accident[Title/Abstract])) OR (acute cerebrovascular accidents[Title/Abstract])) OR (CVA[Title/Abstract])) OR (CVAs[Title/Abstract])) OR (apoplexy[Title/Abstract])) OR (brain attack[Title/Abstract])) OR (ischemic stroke[Title/Abstract])) OR (ischaemic stroke[Title/Abstract])) OR (hemorrhagic stroke[Title/Abstract])) OR (haemorrhagic stroke[Title/Abstract])) OR (cerebral infarction[Title/Abstract])) OR (brain infarction[Title/Abstract])) OR (cerebral ischemia[Title/Abstract])) OR (cerebral ischaemia[Title/Abstract])) OR (brain ischemia[Title/Abstract])) OR (brain ischaemia[Title/Abstract])) OR (cerebral hemorrhage[Title/Abstract])) OR (cerebral haemorrhage[Title/Abstract])) OR (brain hemorrhage[Title/Abstract])) OR (brain haemorrhage[Title/Abstract])) OR (intracerebral hemorrhage[Title/Abstract])) OR (intracerebral haemorrhage[Title/Abstract])) OR (intracranial hemorrhage[Title/Abstract])) OR (intracranial haemorrhage[Title/Abstract])) OR (subarachnoid hemorrhage[Title/Abstract])) OR (subarachnoid haemorrhage[Title/Abstract])) OR

(lacunar stroke[Title/Abstract])) OR (lacunar infarction[Title/Abstract])

#6 #4 OR #5

#7 "Upper Extremity"[Mesh]

#8 (((upper limb[Title/Abstract]) OR (upper extremity[Title/Abstract])) OR (arm[Title/Abstract])) OR (hand[Title/Abstract])

#9 #7 OR #8

#10 randomized controlled trial[Publication Type] OR controlled clinical trial[Publication Type] OR randomized[Title/Abstract] OR randomised[Title/Abstract] OR randomly[Title/Abstract] OR placebo[Title/Abstract]

#11 #3 AND #6 AND #9 AND #10

2. Embase Search Strategy:

#1 'brain computer interface'/exp OR 'brain computer interface'

#2 'brain-computer interface':ab,ti OR 'brain-computer interfaces':ab,ti OR 'BCI':ab,ti OR 'brain-machine interface':ab,ti OR 'brain-machine interfaces':ab,ti OR 'brain machine interface':ab,ti OR 'brain machine interfaces':ab,ti OR 'neural interface':ab,ti OR 'neural interfaces':ab,ti OR 'neural interface technology':ab,ti OR 'neural interface technologies':ab,ti OR 'brain signal interface':ab,ti OR 'brain signal interfaces':ab,ti

#3 #1 OR #2

#4 'cerebrovascular accident'/exp OR 'cerebrovascular accident'

#5 'strokes':ab,ti OR 'acute stroke':ab,ti OR 'acute strokes':ab,ti OR 'stroke':ab,ti OR 'cerebrovascular accidents':ab,ti OR 'acute cerebrovascular accident':ab,ti OR 'acute cerebrovascular accidents':ab,ti OR 'CVA':ab,ti OR 'CVAs':ab,ti OR 'apoplexy':ab,ti OR 'brain attack':ab,ti OR 'ischemic stroke':ab,ti

#6 #4 OR #5

#7 'upper limb'/exp OR 'upper limb'

#8 'upper extremity':ab,ti OR 'arm':ab,ti OR 'hand':ab,ti OR 'shoulder':ab,ti OR 'elbow':ab,ti OR 'wrist':ab,ti

#9 #7 OR #8

#10 'randomized controlled trial'/exp OR 'randomized controlled trial'

#11 'controlled clinical trial':ab,ti OR 'randomized':ab,ti OR 'placebo':ab,ti OR 'RCT':ab,ti OR 'randomised':ab,ti

#12 #10 OR #11

#13 #3 AND #6 AND #9 AND #12

3. Web of Science Search Strategy:

#1 TS=(brain-computer interface OR brain-computer interfaces OR BCI OR brain-machine interface OR brain-machine interfaces OR brain machine interface OR brain machine interfaces OR neural interface OR neural interfaces OR neural interface technology OR neural interface technologies OR brain signal interface OR brain signal interfaces)

#2 TS=(strokes OR acute stroke OR acute strokes OR cerebrovascular accident OR cerebrovascular accidents OR acute cerebrovascular accident OR acute cerebrovascular accidents OR CVA OR CVAs OR apoplexy OR brain attack OR ischemic stroke OR ischaemic stroke OR hemorrhagic stroke OR haemorrhagic stroke OR cerebral infarction OR brain infarction OR cerebral ischemia OR cerebral ischaemia OR brain ischemia OR brain ischaemia OR cerebral hemorrhage OR cerebral haemorrhage OR brain hemorrhage OR brain haemorrhage OR intracerebral hemorrhage OR intracerebral haemorrhage OR intracranial hemorrhage OR intracranial haemorrhage OR subarachnoid hemorrhage OR subarachnoid haemorrhage OR lacunar stroke OR lacunar infarction)

#3 TS=(upper limb OR arm OR hand OR shoulder OR elbow OR wrist)

#4 TS=(randomized controlled trial OR controlled clinical trial OR randomized OR placebo OR RCT OR randomised)

#5 #1 AND #2 AND #3 AND #4

4. Cochrane Library Search Strategy:

#1 MeSH descriptor: [Brain-Computer Interfaces] explode all trees

#2 (brain-computer interface):ti,ab,kw OR (brain-computer interfaces):ti,ab,kw OR

(BCI):ti,ab,kw OR (brain-machine interface):ti,ab,kw OR (brain-machine interfaces):ti,ab,kw OR (brain machine interface):ti,ab,kw OR (brain machine interfaces):ti,ab,kw OR (neural interface):ti,ab,kw OR (neural interfaces):ti,ab,kw OR (neural interface technology):ti,ab,kw OR (neural interface technologies):ti,ab,kw OR (brain signal interface):ti,ab,kw OR (brain signal interfaces):ti,ab,kw

#3 #1 OR #2

#4 MeSH descriptor: [Stroke] explode all trees

#5 (stroke):ti,ab,kw OR (strokes):ti,ab,kw OR (acute stroke):ti,ab,kw OR (acute strokes):ti,ab,kw OR (cerebrovascular accident):ti,ab,kw OR (cerebrovascular accidents):ti,ab,kw OR (acute cerebrovascular accident):ti,ab,kw OR (acute cerebrovascular accidents):ti,ab,kw OR (CVA):ti,ab,kw OR (CVAs):ti,ab,kw OR (apoplexy):ti,ab,kw OR (brain attack):ti,ab,kw OR (ischemic stroke):ti,ab,kw OR (ischaemic stroke):ti,ab,kw OR (hemorrhagic stroke):ti,ab,kw OR (haemorrhagic stroke):ti,ab,kw OR (cerebral infarction):ti,ab,kw OR (brain infarction):ti,ab,kw OR (cerebral ischemia):ti,ab,kw OR (cerebral ischaemia):ti,ab,kw OR (brain ischemia):ti,ab,kw OR (brain ischaemia):ti,ab,kw OR (cerebral hemorrhage):ti,ab,kw OR (cerebral haemorrhage):ti,ab,kw OR (brain hemorrhage):ti,ab,kw OR (brain haemorrhage):ti,ab,kw OR (intracerebral hemorrhage):ti,ab,kw OR (intracerebral haemorrhage):ti,ab,kw OR (intracranial hemorrhage):ti,ab,kw OR (intracranial haemorrhage):ti,ab,kw OR (subarachnoid hemorrhage):ti,ab,kw OR (subarachnoid haemorrhage):ti,ab,kw OR (lacunar stroke):ti,ab,kw OR (lacunar infarction):ti,ab,kw

#6 #4 OR #5

#7 MeSH descriptor: [upper extremity] explode all trees

#8 (upper extremity):ti,ab,kw OR (upper limb):ti,ab,kw OR (arm):ti,ab,kw OR (hand):ti,ab,kw OR (shoulder):ti,ab,kw OR (elbow):ti,ab,kw OR (wrist):ti,ab,kw

#9 #7 OR #8

#10 MeSH descriptor: [randomized controlled trial] explode all trees

#11 (randomized controlled trial):ti,ab,kw OR (controlled clinical trial):ti,ab,kw OR (randomized):ti,ab,kw OR (placebo):ti,ab,kw OR (RCT):ti,ab,kw OR (randomised):ti,ab,kw

#12 #10 OR #11

#13 #3 AND #6 AND #9 AND #12

5. Scopus Search Strategy:

#1 TITLE-ABS-KEY ("brain-computer interface" OR "brain-computer interfaces" OR "BCI" OR "brain-machine interface" OR "brain-machine interfaces" OR "brain machine interface" OR "brain machine interfaces" OR "neural interface" OR "neural interfaces" OR "neural interface technology" OR "neural interface technologies" OR "brain signal interface" OR "brain signal interfaces")

#2 TITLE-ABS-KEY ("strokes" OR "acute stroke" OR "acute strokes" OR "cerebrovascular accident" OR "cerebrovascular accidents" OR "acute cerebrovascular accident" OR "acute cerebrovascular accidents" OR "CVA" OR "CVAs" OR "apoplexy" OR "brain attack" OR "ischemic stroke" OR "ischaemic stroke" OR "hemorrhagic stroke" OR "haemorrhagic stroke" OR "cerebral infarction" OR "brain infarction" OR "cerebral ischemia" OR "cerebral ischaemia" OR "brain ischemia" OR "brain ischaemia" OR "cerebral hemorrhage" OR "cerebral haemorrhage" OR "brain hemorrhage" OR "brain haemorrhage" OR "intracerebral hemorrhage" OR "intracerebral haemorrhage" OR "intracranial hemorrhage" OR "intracranial haemorrhage" OR "subarachnoid hemorrhage" OR "subarachnoid haemorrhage" OR "lacunar stroke" OR "lacunar infarction")

#3 TITLE-ABS-KEY ("upper extremity" OR "upper limb" OR "arm" OR "hand" OR "shoulder" OR "elbow" OR "wrist")

#4 TITLE-ABS-KEY ("randomized controlled trial" OR "controlled clinical trial" OR "randomized" OR "placebo" OR "RCT" OR "randomised")

#5 #1 AND #2 AND #3 AND #4
